# How to improve the clinical diagnosis of acute appendicitis in resource limited settings

**DOI:** 10.1186/s13017-016-0071-8

**Published:** 2016-04-26

**Authors:** Alfredo Alvarado

**Affiliations:** Society of Laparoscopic Surgeons, Miami, FL U.S.A; International College of Surgeons, Chicago, IL USA; American Medical Association, Chicago, IL USA; American College of Emergency Physicians, Irving, TX USA; Pennsylvania Medical Society, Harrisburg, PA USA

**Keywords:** Acute appendicitis, Diagnosis, Alvarado score

## Abstract

This article is a general review of the diagnostic tools that the clinician can use for the early diagnosis of acute appendicitis with emphasis on the Alvarado Score, and it is aimed principally to the medical practitioners in different parts of the world where the diagnostic facilities and technological resources are limited.

## Abstracto (Spanish)

Este artículo es una revisión general de las herramientas diagnósticas que el médico clínico puede usar para el diagnóstico temprano de la apendicitis aguda con énfasis en la escala de Alvarado, y está destinado principalmente a los médicos generales en diferentes partes del mundo donde las ayudas diagnosticas y los recursos tecnológicos son limitados.

## Background

Acute appendicitis is a common cause of abdominal pain in all ages since it occurs in 7 % of the US population and has an incidence of 1.1 cases per 1.000 persons each year. However, it is often a perplexing problem especially during the early stages of the disease that in some cases could delay the diagnosis and could contribute to the persistent rate of morbidity and mortality. The classic signs and symptoms are present in only 60 to 70 % of the cases which indicates the difficulty to ascertain a correct diagnosis. Therefore, the clinician has to improve his diagnostic acumen by looking carefully for those signs and symptoms. When using regular clinical methods, the correct diagnosis can be obtained in between 71 and 97 % of cases, and the rate of negative appendectomies varies between 14 and 75 % [1, 2], and in certain areas of the world, may reach 85 % [3]. The incidence of perforated appendicitis varies between 4 and 45 %, and the death rate ranges from 0.17 to 7.5 % with a peak of 20 % in children under age two [4].

## Clinical method

Here I am presenting my own method for the early diagnosis of acute appendicitis [5] which has been proved to be helpful in many studies around the world. The method relies on a combination of factors derived from physical signs, symptoms, and laboratory tests. The method uses a simple mnemonics (MANTRELS) that is easy to remember and can be applied in many settings without the need of a computer. This mnemonics makes the method even easier to follow: First you check for the three main symptoms: Migration, Anorexia, and Nausea or vomiting; then you follow with the signs which are: Tenderness in the right lower quadrant, Rebound pain (Blumberg sign) and Elevation of the temperature (oral temperature of 37.3 C or more); and finally, you do the lab tests, essentially a CBC to look for Leukocytosis and Shift to the left (increased Stabs or Segmented neutrophils), and a urinalysis to look for acetone as an indication of anorexia if it is not clinically apparent. Rebound pain can be replaced with other indirect signs such as the Rovsing sign and also with the Dunphy’s sign [6] (cough test) or the Markle’s test [7] (heel-drop jarring test). Another indirect sign that can replace the rebound pain is tenderness by gentle direct percussion with the fist, as a mallet, on the right lumbar area in cases of a retrocecal acute appendicitis. In order of decreasing importance the eight best predicting factors proved to be: Localized tenderness on the right lower quadrant, leukocytosis, migration of pain, shift to the left, temperature elevation, nausea and vomiting, anorexia or acetone in the urine and direct rebound pain. I assigned a value of 2 to each of the two of the more important factors tenderness and leukocytosis), and a value of 1 to each one of the others, for a possible total score of 10. A score of 5 or 6 is compatible with the diagnosis of acute appendicitis, a score of 7 or 8 indicates a probable appendicitis, and a score of 9 or 10 indicates a very probable appendicitis. To this score the clinician could subtract 2 points if the patient complains of headache because this symptom is very rare in cases of acute appendicitis. In this particular situation the patient may need further investigation to rule out a different disorder. Scores of five or six are in a grey area, and in this case, you may want to observe the patient for a short time (reevaluate every four to six hours) for 12 or 24 h and if the score stays the same consider other tests such as ultrasound or diagnostic laparoscopy. When the score is three or four, the clinician has two options: This could represent a very early stage of an acute appendicitis so the clinician could keep the patient under observation and repeat the tests, or even more, order additional tests such as an US or CT scan if they are available in that particular setting. Another option is to rely on his clinical impression because, as I already mentioned in my original article, “there is always an intangible ingredient in the diagnosis of acute appendicitis. If there is any question about the diagnosis, more physical examinations and laboratory tests should be performed and the patient should be evaluated every four or six hours, preferably in the hospital”. If the score remains the same or increases after this evaluation, the patient may need surgery. As we can see the diagnosis of acute appendicitis is a dynamic process that go hand to hand with the pathological changes of the disease. As we all know, medicine is a combination of science and art, both of them equally important in the diagnosis of acute appendicitis, so we cannot discard one of them in favor of the other. It is for this reason that we cannot depend on the technological advances only but we should use our common sense and clinical experience to arrive to a correct diagnosis.

When you get to seven, the probability of appendicitis raises dramatically so you may want to operate especially if the patient is a young male. If the patient is a woman, additional investigations may be required to rule out gynecological disorders. This particular situation was validated in a meta-analysis study of adult and pediatric patients which proved that an Alvarado score below four to five rules out the diagnosis of acute appendicitis in children, and in adults, an Alvarado score of eight to nine or higher rules in the diagnosis. The Pediatric Appendicitis Score (PAS) did not identify clinically useful low- or high-risk groups at typical pretest probabilities [8].

The only laboratory tests that are needed in the initial evaluation for acute appendicitis is a complete CBC to determine if there is shift to the left, that is to say, stabs or bands (more than 5 %) or increased segmented neutrophils (more than 75 %) since they are elevated in the early stages of the inflammatory process. Then, several hours later, there is an increase of the whole number of leucocytes and you will find a leukocytosis of more than 10.000/ml. A urinalysis is useful to determine if there is acetone which indicates a fasting state related to anorexia, and also it may show a few red blood cells due to an inflammatory process around the appendix. If the urine shows too many red cells it may point to a ureteral calculus and further investigation should be done. The C-reactive protein test is not enclosed in my score because it is a non-specific test that detects an inflammatory process only and is not diagnostic for any particular condition. Besides this, it would be a redundancy because the shift to the left and leukocytosis are doing the same thing.

## Diagnostic laparoscopy

Diagnostic laparoscopy for suspected appendicitis is recommended for young women, the elderly, or other patients with unclear pathology because of its broader diagnostic ability and for obese patients due to its improved technical use. In one study [9] the Alvarado score combined with selective laparoscopy gave a rate of 0 % cases of negative appendectomy so this approach was recommended for widespread use in the management of suspected acute appendicitis. In other study [10], using the LAPP score the authors found that this score has a high positive and negative predictive value so it can be used by surgeons to evaluate the appendix during diagnostic laparoscopy.

## Ultrasonography

Ultrasound is a widely used technique in the diagnosis of acute appendicitis, however its utilization still remains controversial. In one study [11], ultrasound was found to have an extremely variable accuracy in the diagnosis of acute appendicitis with a sensitivity range from 44 to 100 %, and a specificity range of 47 to 99 %. In another study [12], radiologist-operated ultrasound had inferior sensitivity and inferior positive predictive values when compared with a CT scan, though it was significant faster to perform, and avoided the administration of contras materials. For this reason “a first-pass” approach using US first and then CT, if US is not diagnostic, would be desirable in some institutions. In another study, [13] it was found that clinical evaluation is still paramount to the management of patients with suspected acute appendicitis before considering medical imaging like ultrasonography and computed tomography. Nevertheless, in cases of clinical doubt, ultrasonography may improve the diagnosis and reduces the negative laparotomy rate, and can also be helpful in detecting peri-appendicular abscesses or gynecological diseases [14].

## Computed tomography

Now these days, routine use of computed tomography in the diagnosis of acute appendicitis is highly controversial due to concerns related to the hazards of ionizing radiation and also about its overutilization in clear-cut clinical presentations. The use of CT scans of the abdomen exposes the patients to high doses of radiation which may be the equivalent of 400 chest X rays [15], and this certainly will increase the risk for development of cancer or leukemia. One study suggested that a large proportion of patients who undergo abdominal and pelvic CT scanning receive medically unnecessary multiphase examinations, resulting in substantial excessive radiation exposal. Approximately 3 million scans were performed annually in the United States in 1980, and by 2008, that number had grown to 67 million [16]. This study suggested that a large proportion of patients undergoing abdominal and pelvic CT scanning receive unindicated additional phases that add substantial excess radiation dose with no associated clinical benefit. In a prospective randomized study of clinical assessment versus computed tomography for the diagnosis of acute appendicitis [17], it was found that clinical assessment, unaided by CT scan, reliably identifies patients who need operation for acute appendicitis, and they undergo surgery sooner so routine use of abdominal/pelvic CT is not warranted, and computed tomography should not be considered the standard of care for the diagnosis of acute appendicitis In a prospective study of 1,630 patients with suspected appendicitis [18], it was found that the overall negative appendectomy rate in patients with a CT scan was similar to that in those without (6 % for both groups). In another study [19], it was found that neither CT nor US improves the diagnostic accuracy or the negative appendectomy rate, in fact, they may delay surgical consultation and appendectomy. In atypical cases, one should consider the selective use of diagnostic laparoscopy instead. In a study using the Alvarado score to decide the need to perform a CT scan in cases of suspected acute appendicitis in an ED setting [20], it was found that with a score of 4 to 6 an adjunctive CT scan would be recommended to confirm the diagnosis. If the Alvarado score is 7 or higher, a surgical consultation should be obtained. A computed tomography would not be necessary in patients with an Alvarado score of three or lower. Recently, a prospective comparison of the Alvarado score and CT scan in the evaluation of suspected appendicitis [21] revealed that CT scans are unnecessary in those patients with an AS of 9 and 10 and recommended that an evaluation by CT scan is of value mainly in patients with an Alvarado score of six or less in males, and eight or less in females.

## Unenhanced MRI

Unenhanced magnetic resonance imaging was performed in a group of 85 patients clinically suspected of having acute appendicitis [22] and the results were similar to the Alvarado score but with a lower Sensitivity and lower Negative Predictive Value. However, this test could increase the diagnostic accuracy but it is not available in many hospital settings.

## Reasonable treatment objectives

1. To reduce the rate of negative appendectomies to 10 % or less,
2. To reduce the rate of perforated appendices to 10 % or less

## Treatment

Once the diagnosis of appendicitis has been established, the treatment is surgical, and in men, the most effective treatment is the classic appendectomy through a McBurney incision. This approach is applicable in a great number of developing countries where the technical and economical resources are scarce, but in most of the western and developed countries laparoscopic appendectomy has become the Gold Standard of treatment.

Recently, some physicians have been using a medical treatment with antibiotics for suspected appendicitis. In one study, in Australia [23], suspected cases of acute appendicitis were treated with outpatient antibiotics incorporating the Alvarado score. Patients with a score of one to four received no treatment, patients with a score of five to seven were treated with antibiotics alone, and patients with a score of 8–10 received early surgical treatment. There were two cases of delayed treatment in association with perforation (2/122 = 1.6 %) and of those who had antibiotic treatment, two (2/42 = 4.8 %) required appendectomy. Another study, in Italy [24], revealed that antibiotics for suspected acute appendicitis are effective and may avoid unnecessary appendectomy, reducing operative rate, surgical risks, and overall costs. However, there were recurrences in patients treated with antibiotics (less than 14 %). Another study [25] revealed that antibiotic treatment of patients with uncomplicated acute appendicitis was not shown to be inferior to appendectomy for uncomplicated appendicitis within the first year of observation following initial presentation of appendicitis. In this study, the majority (73 %) of patients with uncomplicated acute appendicitis were successfully treated with antibiotics. These results suggest that patients with CT-proven uncomplicated acute appendicitis should be able to make an informed decision between antibiotic treatment and appendectomy. The problem with this approach is that the patients are being subjected to high doses of ionizing radiation that may develop years later into cancer or leukemia. In a more recent study [26], using a series of meta-analysis, it was found that appendectomy was significantly more efficient than antibiotics alone, and that exclusive antibiotic therapy was associated with a higher rate of readmissions, which varied between 14.2 and 20 %. The conclusion of this study was that appendectomy is considered the gold standard for treating uncomplicated acute appendicitis. Nevertheless, for a selected subgroup of patients with no risk factors for complicated appendicitis, conservative treatment with antimicrobials may be safe and effective.

## Conclusion

Diagnosis of acute appendicitis can be improved if the clinician uses a careful history and physical examination, and simple laboratory tests. However, under certain circumstances, additional tests could be needed. This approach has given good results in various studies around the world and proves that the Alvarado score is a simple, practical, economical and reliable method for the diagnosis of acute appendicitis.
